# Oculomotor atypicalities in motor neurone disease: a systematic review

**DOI:** 10.3389/fnins.2024.1399923

**Published:** 2024-06-26

**Authors:** Megan Rose Readman, Megan Polden, Melissa C. Gibbs, Aisling Donohue, Suresh K. Chhetri, Trevor J. Crawford

**Affiliations:** ^1^Department of Psychology, Lancaster University, Lancaster, United Kingdom; ^2^Department of Primary Care and Mental Health, The University of Liverpool, Liverpool, United Kingdom; ^3^National Institute of Health Research Applied Research Collaboration North West Coast, Liverpool, United Kingdom; ^4^Division of Health Research, Lancaster University, Lancaster, United Kingdom; ^5^Nuffield Department of Clinical Neurosciences, John Radcliffe Hospital, University of Oxford, Oxford, United Kingdom; ^6^School of Psychology, Faculty of Health, Liverpool John Moores University, Liverpool, United Kingdom; ^7^Lancashire and South Cumbria Motor Neurone Disease Care and Research Centre, Neurology Department, Lancashire Teaching Hospitals NHS Foundation Trust, Royal Preston Hospital, Preston, United Kingdom

**Keywords:** motor neurone disease, saccades, prosaccade, antisaccade, smooth pursuit, memory guided saccade

## Abstract

**Introduction:**

Cognitive dysfunction is commonplace in Motor Neurone Disease (MND). However, due to the prominent motor symptoms in MND, assessing patients’ cognitive function through traditional cognitive assessments, which oftentimes require motoric responses, may become increasingly challenging as the disease progresses. Oculomotor pathways are apparently resistant to pathological degeneration in MND. As such, abnormalities in oculomotor functions, largely driven by cognitive processes such as saccades and smooth pursuit eye movement, may be reflective of frontotemporal cognitive deficits in MND. Thus, saccadic and smooth pursuit eye movements may prove to be ideal mechanistic markers of cognitive function in MND.

**Methods:**

To ascertain the utility of saccadic and smooth pursuit eye movements as markers of cognitive function in MND, this review summarizes the literature concerning saccadic and smooth pursuit eye movement task performance in people with MND.

**Results and discussion:**

Of the 22 studies identified, noticeable patterns suggest that people with MND can be differentiated from controls based on antisaccade and smooth pursuit task performance, and thus the antisaccade task and smooth pursuit task may be potential candidates for markers of cognition in MND. However, further studies which ascertain the concordance between eye tracking measures and traditional measures of cognition are required before this assumption is extrapolated, and clinical recommendations are made.

**Systematic review registration:**

https://www.crd.york.ac.uk/prospero/display_record.php?RecordID=376620, identifier CRD42023376620.

## 1 Introduction

Motor neurone disease (MND) is an adult-onset fatal neurodegenerative disorder, of unknown etiology, characterized by the progressive degeneration of motor neurons in the primary motor cortex, corticospinal tracts, brainstem, and spinal cord (Logroscino et al., 2008). The clinical presentation results from progressive wasting and weakness of the bulbar, limb and respiratory muscles (Foster and Salajegheh, 2019). The condition can be sporadic or familial and demonstrates marked phenotypic heterogeneity (Foster and Salajegheh, 2019). Amyotrophic Lateral Sclerosis (ALS) is the most common phenotype and involves degeneration of both upper and lower motor neurones (Masrori and Van Damme, 2020). Patients with primary lateral sclerosis (PLS) have predominantly upper motor neuron dysfunction (Turner et al., 2020), while those with progressive muscular atrophy (PMA) have lower motor neuron dysfunction (Visser et al., 2008). Recent studies indicate the global incidence of MND to be between 0.6 and 3.8 per 100,000 person-years (Longinetti and Fang, 2019), with a slightly elevated incidence rate of between 2.1 and 3.8 per 100,000 person-years in Europe (Longinetti and Fang, 2019).

Motor neurone disease is a clinical diagnosis and investigations help to exclude alternative causes, but nerve conduction studies/electromyography can support the diagnosis. Cognitive deficits (in particular attentional, verbal fluency, working memory, planning and mental shifting deficits (Lillo and Hodges, 2010), are increasingly recognized as common place in MND (Phukan et al., 2007; Burrell et al., 2016). Current evidence indicates that between 28 and 50% of MND patients present with cognitive deficits indicative of frontotemporal dysfunction (Rakowicz and Hodges, 1998; Portet et al., 2001; Ringholz et al., 2005; Chiò et al., 2019) with ∼13- 23% of patients satisfying the criterion for frontotemporal dementia (Rippon et al., 2006; Phukan et al., 2012; Chiò et al., 2019). Moreover, cognitive deficits are thought to increase with advancing disease stage (Crockford et al., 2018).

Eye movement disorders, including saccadic abnormalities, are recognized in a number of neurodegenerative conditions. Saccades are rapid ballistic eye movements that abruptly shift the point of fixation from one part of a given visual field to another (Westheimer, 1954; Singh and Singh, 2012). Saccades are thought to depend upon multiple neuronal pathways controlled by cortical networks in the frontal lobe, parietal lobe and downstream pathways that project to the cerebellum and brainstem (Anderson and MacAskill, 2013). Importantly these brain regions are implicated in several cognitive operations, in particular spatial attention (Deubel and Schneider, 2003), working memory (Stuss and Benson, 2019) and planning (Faglioni, 2020). Therefore, saccadic eye movements (SEMs) serve as a useful indicator of specific cognitive functioning. Indeed, SEM abnormalities are sensitive markers of cognitive function in clinical disorders including progressive neurodegenerative disorders such as Alzheimer’s disease (Crawford et al., 2005, 2013; Anderson and MacAskill, 2013) and Parkinson’s disease (Crawford et al., 2002; MacAskill et al., 2012; Anderson and MacAskill, 2013; Antoniades et al., 2015), and psychiatric disorders including Schizophrenia (Diefendorf and Dodge, 1908; Bittencourt et al., 2013).

Smooth pursuit eye movements enable the tracking of moving objects in the visual scene. Such tracking is obtained through a combination of smooth movements and saccades, which serve to realign the object should it fall outside the fovea, the area of highest acuity (Barnes, 2008). Smooth pursuit eye movements are typically assumed to be voluntary and depend upon cognitive operations including selection, learning, prediction, and attention to environmental motion (Barnes, 2008). Therefore, smooth pursuit eye movements may also serve as an indicator of cognitive function, although potentially to a lesser extent than saccades. Indeed, characteristic atypicalities in smooth pursuit eye movements have been observed in patients with Alzheimer’s disease (Molitor et al., 2015) and Parkinson’s disease (Frei, 2021).

**FIGURE 1:**
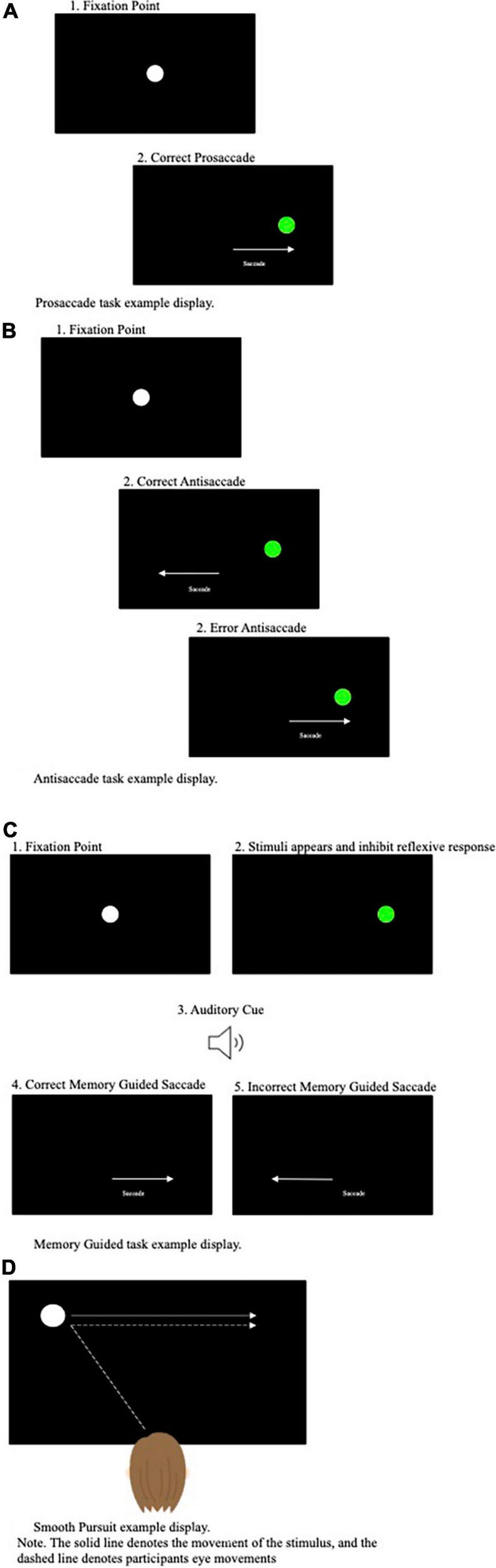
**(A)** Prosaccade task example display. **(B)** Antisaccade task example display. **(C)** Memory guided task display. **(D)** Smooth pursuit example display.

Eye tracking is a non-invasive advanced technology that provides reliable multifaceted measures of an individual’s saccades and smooth pursuit eye movements whilst performing tasks. Whilst a plethora of eye tracking tasks have been developed, several tasks have been adopted far more frequently than others. These tasks include: (1) the prosaccade task, (2) the antisaccade task, (3) the memory guided saccade task, and (4) the smooth pursuit tasks (see Figure 1 for pictural representation of these tasks).

The prosaccade task, perhaps the most simplistic of the often-used eye tracking tasks, requires participants to perform rapid, reactive saccades toward a suddenly appearing target from a central fixation point (see Figure 1A). The antisaccade task (Hallett, 1978) in comparison is more cognitively demanding and requires participants to perform arguably counterintuitive eye movements. Specifically, during the antisaccade task, a target is presented in the participant’s peripheral visual field and participants are asked to direct their gaze to the opposite side to the target’s location (Munoz and Everling, 2004) (see Figure 1B). When a target is presented in an individual’s visual field, the reflexive response is to perform a prosaccade toward the target. Subsequently, it has been proposed that successful performance on the antisaccade task relies upon executive functioning, attentional processes and inhibitory control processes (i.e., inhibiting the natural response to look toward the target) (Hutton and Ettinger, 2006). As a result, the antisaccade task has been assumed to be a powerful measure of cognitive functioning, in particular inhibitory control processes.

The memory guided, or remembered, saccade task targets overlapping cognitive operations to the antisaccade task in terms of requiring response inhibition. Specifically, the memory guided saccade task requires the remembrance of a peripheral target location, whilst inhibiting the urge to make a saccade ahead the offset of the central fixation point and an auditory cue (see Figure 1C). Thus, this paradigm is thought to examine the inhibitory processes (the inhibition of a reflexive prosaccade action) and spatial working memory (the ability to generate an internal representation of space) (Smith and Crawford, 2021).

The prosaccade, antisaccade and memory guided saccade tasks require shifts of attention from a fixation point to a target control by overlapping cortical-collicular projection. In contrast, the smooth pursuit task requires focused attention on the target on modulation of eye-velocity to match that of the target. Therefore, rendering the smooth pursuit task entirely different both in terms of function and the neural systems recruited during execution. During the smooth pursuit task participants are asked to track a moving object in their visual display as smoothly as possible. Dissimilar to SEMs, that are primarily directed toward stationary objects, smooth pursuit eye movements require slower tracking eye movements aiming to keep the moving object on the fovea (see Figure 1D).

Classically, with the exception of some extreme patients, the oculomotor pathways are assumed to be spared in MND (Nijssen et al., 2017). As such, SEM deficits observed in those with MND may perhaps be reflective of the frontotemporal cognitive deficits observed in MND rather than alterations in the oculomotor neurons themselves. In particular, increased error rate (Sharma et al., 2012; Proudfoot et al., 2016) and latency have been observed on in people with MND on the anti-saccade task (Proudfoot et al., 2016).

Despite the promise of saccades and smooth pursuit eye movements in providing an indicator of cognitive function in people with MND, research in this area remains limited and underdeveloped. Subsequently, this review aims to summarize the latest developments in the literature concerning SEM task performance in people with MND compared to healthy controls.

## 2 Methods

### 2.1 Protocol preregistration

This review was conducted in accordance with the Preferred Reporting Items for Systematic Reviews and Meta-Analyses (PRISMA) (Page et al., 2021) guidelines and was pre-registered on PROSPERO (ID: CRD42022370067).

### 2.2 Search protocol

A comprehensive literature search was conducted on 5th December 2022, using Academic Search Ultimate, CINHAL and MEDLINE, and Web of Science and Scopus. These databases, accessed through Lancaster University, were selected to address the multidisciplinary nature of the research question.

Two independent search strings, one pertaining to the population (MND) and one pertaining to eye movement tasks, of interest were developed. These two search strings were then combined with the ‘AND’ operator. The search strings applied to each database differed only in database specific dictionary terms included. Otherwise, the free-text search terms remained consistent. Appropriate free-text search terms were identified during scoping searches (see Appendix 1 for full search strings applied).

The sensitivity of our search strings was assessed through “search strategy testing” on each of the selected databases. To do so, we checked that our searches found three highly relevant papers that were identified during scoping searches. The following papers were used for this test: Sharma et al. (2012), Proudfoot et al. (2016), and Guo et al. (2022). The initially developed search strings proved to be sensitive (i.e., found all four papers), and so were not updated.

To ensure that relevant records published after the point of running the initial searches on 5th December 2022 were captured, we re-ran our searches on 30th November. To ensure that all relevant records were captured, we completed forward and backward citation tracking of included papers using Google Scholar. Moreover, to reduce the impact of publication bias on our review, we searched for relevant pre-prints using PsyArxiv, MedArxiv, and bioArxiv. Pre-prints were searched using combinations of the following search terms: (”motor neuron disease” OR “amyotrophic lateral sclerosis”) AND (”eye movements” OR “saccades”). Records that appeared relevant from the abstract were subjected to full-text screening.

The .ris files downloaded during the final search of each database were exported into CADIMA, an online open-access tool designed to facilitate each stage of conducting a systematic review (Kohl et al., 2018). The records were automatically de-duplicated using CADIMA’s de-duplication tool and were double-checked by hand. Of the 4,032 records identified, 817 (20.26%) of these were duplicates resulting in 3,215 articles eligible for screening. In circumstances in which we could not access the relevant record, Lancaster University Library requested access to these records.

### 2.3 Inclusion criterion

Screening was conducted in two phases: (1) title and abstract screening and (2) full text screening. The criteria applied during title and abstract screening were: (1) the abstract is written in the English language, (2) this is an original piece of research, (3) the study involves human participants, (4) there is mention of oculomotor function or eye tracking technology (or related terms), and (5) there is mention of Motor Neuron(e) or related terms. The criteria applied to full text screening were: (1) the study involves a saccadic/smooth pursuit eye movement task, (2) a healthy control condition is included, (3) the study includes an MND group free from disclosed comorbidities, and (4) an experimental or pre-post design is used (no case studies).

It is extensively documented that various clinical conditions, including but not limited to neurodegenerative disorders [e.g., Alzheimer’s disease and Parkinson’s Disease (Anderson and MacAskill, 2013)], psychiatric disorders [e.g., anxiety disorders (Ainsworth and Garner, 2013), obsessive compulsive disorder (Bey et al., 2018), Schizophrenia (Bittencourt et al., 2013)], and neurodevelopmental disorders [e.g., Autism spectrum disorder (Caldani et al., 2020)], are associated with alternations in saccadic and smooth pursuit eye movements. The occurrence of comorbid psychiatric impairments is increasingly being recognized as prevalent in MND (Patten et al., 2007). Given psychiatric impairments can independently influence saccadic and smooth pursuit metrics individuals, papers that recruited only MND patients with disclosed comorbidities were excluded.

### 2.4 Screening

Two raters (MRR, MP or AD) independently screened all records during both stages of screening. The Inter-rater reliability of title and abstract screening was assessed and deemed to be “Good” (*k* = 0.69) by CADIMA for a small portion of records (10%) prior to screening any further records.

**FIGURE 2:**
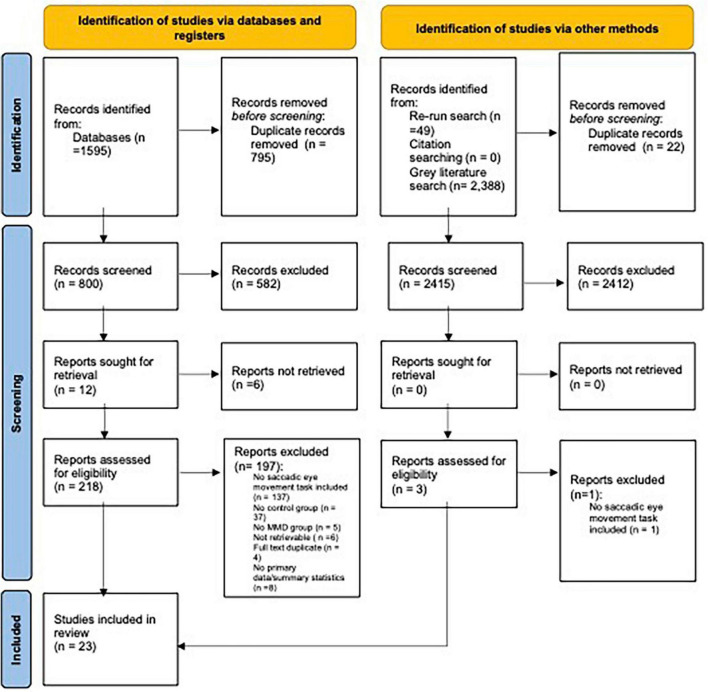
PRISMA flowchart: outlining number of papers excluded at each stage of screening. Only the first reason for exclusion is reported. From Page et al. (2021).

The level of inter-rate agreement for full text screening was also deemed to be “Good” (*k* = 0.75). When inconsistencies in rating arose, a third reviewer was involved in making the final decision. Of the 800 full-text records screened, 780 (97.5%) were excluded due to failure to meet our inclusion criteria (see Figure 2 for full breakdown for reason for exclusion). All records that passed the full-text screening phase were checked for retraction using the Retraction Watch Database (22 November 2023),^1^ with none of the records being flagged as being retracted.

### 2.5 Data extraction

Data was extracted by a single reviewer (either MRR or MP) and was checked by the other reviewer to ensure no missing data and the data extracted was correct. The following data was extracted: participant groups; the number of participants per participant group; mean age of each participant group; diagnostic criterion applied; MND clinical subtype; MND age at onset; MND duration; MND site of onset; MND Progression Rate; MND severity; MND Survival; cognitive tests; eye tracking tasks employed; eye tracking device; direction of the main effects; any reported means and standard deviations (SDs); and any relevant conclusions made by the authors (see Supplementary Table 1 for data extraction table).

### 2.6 Quality assessment

To provide an indication of the credence that should be given to individual studies we assessed the methodology quality of the papers that were analyzed. Methodological quality was assessed using the Mixed Methods Appraisal Tool (Hong et al., 2018), a tool which is suitable for evaluating methodological quality across heterogeneous designs. MRR and MP quality assessed all papers independently with excellent levels of agreement (95.7% agreement). Inconsistencies were resolved during discussions between both reviewers (see Supplementary Table 2 for full quality assessment).

## 3 Results

### 3.1 Characteristics of included studies

A total of 23 papers met the inclusion criterion. All studies recruited an MND group and a control group free from any disclosed clinical condition that may affect eye movements. The majority (*N* = 11) of studies exclusively recruited people with ALS (Shaunak et al., 1995; Evdokimidis et al., 2002; Moss et al., 2012; Burrell et al., 2013; Witiuk et al., 2014; Gorges et al., 2015; Becker et al., 2019; Yunusova et al., 2019; Guo et al., 2022; Rekik et al., 2022; Raveh et al., 2023; Riek et al., 2023; Zaino et al., 2023), two studies recruited people with ALS and people with Primary Lateral Sclerosis (PLS) (Sharma et al., 2012; Proudfoot et al., 2016), two studies recruited people with ALS, progressive muscular atrophy (PMA) or PLS (Marti-Fàbregas and Roig, 1993; Poletti et al., 2021), three studies did not disclose the MND subtype (Abel et al., 1992, 1995; Donaghy et al., 2010), one study recruited people with spinal muscular atrophy (SMA) only (Anagnostou et al., 2021), one study recruited people with Kennedy’s disease (Anagnostou et al., 2019), and the final study recruited people with ALS and progressive bulbar palsy (Esteban et al., 1978).

In most studies (*N* = 10) MND was diagnosed in accordance with the Revised El Escorial criteria; however two studies applied the World Federation of Neurology diagnostic criterion, one study relied upon genetic testing, one study relied upon Electromyography and muscle biopsy analysis and the remaining six studies did not report the diagnostic criterion adhered to. Sample sizes in each study varied from as few as 7 to 864 people with MND (*M* = 77.86(182.2), Median = 32). A total of 1,670 people with MND (*M* = 61.85(123.48) and 1,419 controls were included across all studies. See Supplementary Table 1 for sample specifications.

The method of eye tracking applied substantially differed across studies with some (*N* = 2) employing bedside eye tracking techniques (i.e., clinical observation without an eye tracking device) and some relying on video/laptop camera recordings (*N* = 2). However, the majority of studies (*N* = 19) relied upon a video-based system that uses infrared light tracking video cameras (e.g., EyeSeeCam, Eyelink and Skalar Medical Iris Camera) (note the device was not recorded in one study).

Studies employed either one or a combination of the following tasks; (1) prosaccade task, (2) antisaccade tasks, (3) memory guided saccade task, and (4) smooth pursuit task. Successful completion of such tasks is thought to recruit distinct oculomotor pathways. Subsequently, following a general overview of the occurrence of oculomotor abnormalities the results obtained across the 21 studies will be presented by oculomotor task.

### 3.2 Occurrence of oculomotor abnormalities

When considering the occurrence of oculomotor abnormalities, Poletti et al. (2021) concluded that oculomotor abnormalities could be detected at bedside examination in 10.5% of people with ALS studied. Moreover, employing videooculographic recording, Rekik et al. (2022) observed abnormalities in eye movement recordings in 72.6% of people with ALS.

When considering whether the occurrence of oculomotor abnormalities differs as a consequence of clinical presentation, Poletti et al. (2021) observed that oculomotor abnormalities were more common in people with bulbar onset compared to spinal onset illness. Moreover, Poletti et al. (2021) also observed that oculomotor abnormalities were more common with increasing disease severity (as quantified by the King’s staging system) and were more common in people with cognitive impairment. Interestingly, people who presented with oculomotor abnormalities typically were older at the point of ALS onset than people who did not present with oculomotor abnormalities (Poletti et al., 2021; Rekik et al., 2022). Thus, taken as a whole, Poletti et al. (2021) concluded that oculomotor atypicalities are a highly specific (94.5%), but less sensitive (35.0%), proxy for cognitive impairment in ALS.

### 3.3 Prosaccade task

Most studies (*n* = 19) employed the prosaccade task (see Table 1 for breakdown of observations by study). A substantial proportion of these studies observed that performance on the prosaccade task did not significantly differ between people with MND and healthy controls (Donaghy et al., 2010; Moss et al., 2012; Sharma et al., 2012; Burrell et al., 2013; Proudfoot et al., 2016; Anagnostou et al., 2019, 2021; Guo et al., 2022; Riek et al., 2023). The typicality of people with MND’s prosaccades was evident at the level of infrared tracking and video recordings. In particular, using video camera recordings Moss et al. (2012) observed that the number of vertical and horizontal prosaccade errors, the number of absent saccades (i.e., no movement when target provided), and number of anticipatory vertical and horizontal prosaccades made by people with ALS did not differ from that of controls.

**TABLE 1:** Prosaccade deficits broken down by metric in MND group level (note, breakdown based on clinical facets is not detail here).

	Change in MND group metrics compared to controls		
Study	Overall occurrence of atypicalities	Latency	Velocity
Sharma et al., 2012	ND		
Proudfoot et al., 2016		ND	ND
Guo et al., 2022		ND	ND
Moss et al., 2012	ND		
Zaino et al., 2023		Prolonged	
Gorges et al., 2015		Prolonged	Decreased
Evdokimidis et al., 2002		Reduced	
Burrell et al., 2013		ND	ND
Shaunak et al., 1995		Prolonged	ND
Rekik et al., 2022		Prolonged	
Witiuk et al., 2014		Reduced	ND
Riek et al., 2023		ND	ND
Raveh et al., 2023			Increased
Poletti et al., 2021	More Common		
Marti-Fàbregas and Roig, 1993	ND	Prolonged	ND
Donaghy et al., 2010	ND		
Anagnostou et al., 2021	ND		ND
Anagnostou et al., 2019	ND		
Esteban et al., 1978	ND		

ND denotes no statistically significant difference.

However, some inconsistencies were noted. For example, Burrell et al. (2013), Proudfoot et al. (2016), and Guo et al. (2022) observed that prosaccade latency did not differ between people with ALS and controls. In contrast, Marti-Fàbregas and Roig (1993), Shaunak et al. (1995), Gorges et al. (2015), Rekik et al. (2022), and Zaino et al. (2023) observed that prosaccade latency was prolonged in people with MND (ALS, PLS and PMA patients) compared to controls. Alternatively, both Evdokimidis et al. (2002) and Witiuk et al. (2014) observed that people with ALS prosaccade latencies were significantly shorter than controls, with Witiuk et al. (2014) observing that the responses of many people with ALS fell within the express saccade epoch.

Concerning saccadic velocity, Shaunak et al. (1995), Burrell et al. (2013), Witiuk et al. (2014), Proudfoot et al. (2016), Anagnostou et al. (2021), Guo et al. (2022), and Riek et al. (2023) observed that peak velocity did not differ between people with SMA and ALS, and controls. In comparison Gorges et al. (2015), observed that peak velocities, especially in horizontal and upper directions, were significantly decreased in people with ALS compared to controls. Furthermore, Raveh et al. (2023) observed that peak velocity was increased in people with ALS compared to controls.

Atypicalities in prosaccades have frequently been observed in people with Alzheimer’s disease (Crawford et al., 2005, 2013; Anderson and MacAskill, 2013). Interestingly, Poletti et al. (2021) observed prosaccade dysfunction was significantly more common in people with ALS (occurring in 2% of the studied cases) compared to controls (*p* < 0.001). However, the frequency of prosaccade dysfunction did not differ between people with ALS and Alzheimer’s disease.

Importantly, whilst there are key differences in the MND phenotype sampled across studies (e.g., ALS vs PLS vs SMA), the discrepancies in observed results do not directly map to these differences in the MND phenotype. Therefore, it seems unlikely that the discrepancies across papers are due to differences in clinical populations sampled. Further supporting this assumption, there is a lack of clear consensus regarding the influence of clinical manifestation and disease severity on prosaccade metrics. For example, both Burrell et al. (2013) and Guo et al. (2022) observed that prosaccade parameters were comparable between people with bulbar and spinal onset. In comparison, Donaghy et al. (2010), Gorges et al. (2015), Raveh et al. (2023), and Zaino et al. (2023) observed that site of ALS onset significantly influenced prosaccade profile. Specifically, bulbar onset was associated with reduced prosaccade velocity (Zaino et al., 2023), increased prosaccade latency (Donaghy et al., 2010), increased prosaccade gain (Raveh et al., 2023), increased prosaccade duration (Zaino et al., 2023) and hypometric prosaccades in the upward direction (Gorges et al., 2015). In comparison, Zaino et al. (2023) observed that people with spinal onset produced vertical and horizontal prosaccades of reduced amplitude compared to bulbar onset patients. Furthermore, Esteban et al. (1978) observed that all people who displayed atypical vertical prosaccades had clinical evidence of severe pseudobulbar damage (quadriplegic). Concerning disease duration, Zaino et al. (2023) observed that the peak velocity of vertical prosaccades was inversely correlated with disease duration in people with bulbar onset, and Marti-Fàbregas and Roig (1993) observed that prosaccade latency decreased as the time from first symptom increased. This overall lack of clear consensus regarding the occurrence of prosaccade deficits in MND patients, and the influence of clinical manifestation and disease severity on prosaccade metrics may perhaps suggest that the prosaccade task lacks sufficient sensitivity to differentiate MND patients from healthy controls.

### 3.4 Antisaccade task

Fourteen studies employed the antisaccade task (see Table 2 for breakdown of observations by study). Consistent with prior literature considering alternative neurological conditions [e.g., Parkinson’s Disease (see Waldthaler et al., 2021, for review] and Alzheimer’s Disease (see Kahana Levy et al., 2018, for review), all but two studies, that measured error rate, demonstrated that the antisaccade error rate is significantly increased in both ALS and PLS compared to controls (Shaunak et al., 1995; Sharma et al., 2012; Witiuk et al., 2014; Gorges et al., 2015; Proudfoot et al., 2016; Becker et al., 2019; Yunusova et al., 2019; Rekik et al., 2022; Raveh et al., 2023; Zaino et al., 2023). In the two studies that did not observe elevated antisaccade error rates, Moss et al. (2012), and Riek et al. (2023) found antisaccade error rate to be comparable between people with ALS and controls. Considering the eye tracking techniques employed, Moss et al. (2012) analyzed antisaccades using bedside tracking techniques (video camera recordings). Thus, it may be that discrepancies between the observations of Moss et al. (2012) and all other antisaccade studies, that measured error rate, are attributable to differences in methodological sensitivity. It is, however, important to note that Yunusova et al. (2019) observed elevated antisaccade error rates in people with ALS when recording eye movements through a laptop camera. Moreover, Riek et al. (2023) observed comparable percentages of antisaccade direction errors when recording eye movements using an infrared light tracking device (Eyelink 1000); thus, it appears unlikely that this discrepancy is due to methodological facets.

**TABLE 2:** Antisaccade deficits broken down by metric in MND group level (note, breakdown based on clinical facets is not detail here).

	Change in MND group metrics compared to controls	
Study	Error rate	Latency
Sharma et al., 2012	Increased	Increased
Proudfoot et al., 2016	Increased	Increased
Yunusova et al., 2019	Increased	
Moss et al., 2012	ND	
Zaino et al., 2023	Increased	
Gorges et al., 2015	Increased	
Evdokimidis et al., 2002		Latency of error antisaccades shorter; latency of correct antisaccades NS
Becker et al., 2019	Increased	
Shaunak et al., 1995	Increased	Increased
Rekik et al., 2022	Increased	
Witiuk et al., 2014	Increased	
Riek et al., 2023	ND	
Raveh et al., 2023	Increased	
Donaghy et al., 2010		Increased

ND denotes no statistically significant difference.

Furthermore, a global increase in antisaccade latency was observed in people with ALS and PLS compared to controls (Shaunak et al., 1995; Donaghy et al., 2010; Sharma et al., 2012; Proudfoot et al., 2016). Importantly, Shaunak et al. (1995), Donaghy et al. (2010), Sharma et al. (2012), and Proudfoot et al. (2016) analyzed the latency of both correct and incorrect antisaccades as one variable. Interestingly, when analyzing the latency of correct and incorrect antisaccades independently, Evdokimidis et al. (2002) observed that correct antisaccades latency did not differ between people with ALS and controls, but the latency of error antisaccades was significantly shorter in people with ALS compared to controls. Thus, suggesting that deficits in inhibitory control (i.e., inhibiting the reflexive prosaccade response) may be influencing the latency of people with ALS’s antisaccades. Supporting this assumption, Witiuk et al. (2014) observed that the percentage of antisaccade errors made by people with ALS increased with increasing cognitive dysfunction, as indicated by decreasing Montreal cognitive assessment score. Moreover, degraded performance on the antisaccade task has been linked to volume reduction in the dorsolateral prefrontal cortex (Yunusova et al., 2019), a brain region frequently implicated in attention and working memory (Katsuki and Constantinidis, 2012; Barbey et al., 2013).

Considering the influence of clinical subtype on antisaccade metrics, both Sharma et al. (2012) and Proudfoot et al. (2016) observed that people with PLS made significantly more antisaccade errors and produced antisaccades of longer latencies than people with ALS, thus indicating that antisaccade metrics may be sensitive to differences in MND subtype. Moreover, within the ALS subtype, people with bulbar onset were both slower to initiate antisaccades (Proudfoot et al., 2016) and made more anti-saccade errors compared to those with limb onset (Proudfoot et al., 2016; Raveh et al., 2023). However, this observation was not consistent with Donaghy et al. (2010) and Zaino et al. (2023), both observing that there were no significant differences in antisaccade error rate between people with bulbar and spinal onset. Therefore, whilst antisaccade metrics may be sensitive to differences in MND subtype (e.g., ALS v PLS) they may not be sufficiently sensitive to more nuanced differences within each subtype (e.g., Bulbar v Spinal onset ALS).

There appears to be a lack of consensus regarding the influence of disease severity on antisaccade metrics. Specifically, Proudfoot et al. (2016) observed that disease severity, quantified by the ALSFRS-R score, but not disease duration or rate of progression, were related to antisaccade error rate. In comparison Zaino et al. (2023) observed that the number of correctly executed antisaccades was inversely correlated with disease duration.

### 3.5 Memory guided saccades

Four studies employed the memory guided saccade task (see Table 3 for breakdown of observations by study). In doing so, it was observed that compared to controls, people with ALS made significantly fewer correct saccades (Gorges et al., 2015; Becker et al., 2019; Zaino et al., 2023) and made significantly fewer corrections of erroneous saccades (Zaino et al., 2023). While Evdokimidis et al. (2002) did not comment on the error rate on the memory guided saccade tasks in people with ALS compared to controls, Evdokimidis et al. (2002) observed that the latency of remembered saccades were significantly longer in ALS patients compared to controls.

**TABLE 3:** Memory guided saccade deficits broken down by metric in MND group level (note, breakdown based on clinical facets is not detail here).

	Change in MND group metrics compared to controls	
Study	Error rate	Latency
Zaino et al., 2023	Increased	
Gorges et al., 2015	Increased	
Evdokimidis et al., 2002		Increased
Becker et al., 2019	Increased	

Zaino et al. (2023) further explored whether specific clinical facets significantly influenced memory guided saccade metrics. In doing so, Zaino et al. (2023) observed that people with bulbar onset ALS made significantly more erroneous memory guided saccades, and in people with spinal onset illness, the percentage of correctly executed saccades positively correlated with parietal cortical gray matter volume. As only one study has sought to elucidate the role of clinical facets in memory guided saccade metrics, these observations should be treated with caution.

### 3.6 Smooth pursuit task

Ten studies employed a smooth pursuit task (see Table 4 for breakdown of observations by study). In line with prior literature which has consistently documented smooth pursuit abnormalities in people with alternative neurological conditions [e.g., Parkinson’s disease (Frei, 2021) and Alzheimer’s disease (Molitor et al., 2015), all bar one study observed substantial group level deficits in smooth pursuits in people with MND compared to controls (Abel et al., 1992, 1995; Marti-Fàbregas and Roig, 1993; Donaghy et al., 2010; Moss et al., 2012; Poletti et al., 2021; Guo et al., 2022; Rekik et al., 2022; Raveh et al., 2023)]. Poletti et al. (2021) and Rekik et al. (2022) observed smooth pursuit abnormalities in 6.9 and 56.5% of people with patients sampled, respectively. Interestingly, the one study, Gorges et al. (2015) that did not observe group level deficits in smooth pursuit eye movements, did however, observe atypicalities at the individual level.

**TABLE 4:** Smooth Pursuit deficits broken down by metric in MND group level (note, breakdown based on clinical facets is not detail here).

	Change in MND group metrics compared to controls				
Study	Overall occurrence of atypicalities	Gain	Prevalence of cogwheeling	Prevalence of saccadic intrusions	Fixation time
Guo et al., 2022		ND	Increased		
Moss et al., 2012				Increased	
Gorges et al., 2015				Increased	
Rekik et al., 2022	Increased				
Raveh et al., 2023					Increased
Poletti et al., 2021	Increased				
Marti-Fàbregas and Roig, 1993		Increased			
Donaghy et al., 2010		Increased			
Abel et al., 1995		Increased			
Abel et al., 1992		Increased			

When breaking down smooth pursuit metrics by type compared to controls, smooth pursuits in people with MND were characterized by increased frequency of saccadic intrusions (anticipatory saccades and square wave jerks) (Abel et al., 1992; Moss et al., 2012; Gorges et al., 2015) reduced gain (Abel et al., 1992, 1995; Marti-Fàbregas and Roig, 1993; Donaghy et al., 2010) and abnormal cogwheel eye movements (the interruption of smooth pursuit movements by catch-up saccades, resulting in jerky, uneven eye movement) (Gorges et al., 2015; Guo et al., 2022). It is, however, important to note that not all abnormalities were consistently reported. Specifically, whilst Abel et al. (1992), Marti-Fàbregas and Roig (1993), Abel et al. (1995), and Donaghy et al. (2010) observed reduced gain in people with MND (ALS and MND non-specified), Guo et al. (2022) reported that gain was comparable between people with ALS and controls.

When considering the influence of disease progression and severity on smooth pursuit metrics, Marti-Fàbregas and Roig (1993) observed that the number of saccadic intrusions increased with rate of progression. Moreover, Abel et al. (1992) observed that smooth pursuit gain was significantly more reduced, in response to increase in stimulus speed, in people with severe MND compared to people with moderate MND. Regarding the influence of clinical presentation on smooth pursuit metrics, Guo et al. (2022) observed that the occurrence of abnormal cogwheel smooth pursuit eye movements was more common in people with ALS who display bulbar involvement than people who do not display bulbar involvement. Comparably, Donaghy et al. (2010) observed that velocity gain was reduced to a greater extent in bulbar onset patients compared to spinal onset patients. Rekik et al. (2022) observed that the occurrence of abnormal smooth pursuits (i.e., smooth pursuits with a gain <0.8 at 3 Hz) was correlated with cognitive dysfunction, as classified by the Mini Mental State Examination (Folstein et al., 1983) and the Frontal Assessment Battery (Dubois et al., 2000). Some prior research (Morimoto et al., 2012) indicates people who present with bulbar-onset and bulbar involvement may be more susceptible to cognitive impairment. Thus, accumulatively the findings of Marti-Fàbregas and Roig (1993), Guo et al. (2022), and Rekik et al. (2022) may potentially indicate that the degree of bulbar involvement influences smooth pursuit deficits in people with ALS.

## 4 Discussion

Despite diagnostic emphasis on motor impairments, it is now widely accepted that cognitive dysfunction is commonplace in MND (Phukan et al., 2007, 2012). As the disease progresses, and the ability to perform motor tasks such as speaking, writing, and drawing, becomes more difficult, assessing patients’ cognitive function through traditional cognitive assessments [e.g., the Montreal Cognitive Assessment (Nasreddine et al., 2005), Edinburgh Cognitive and Behavioral ALS Screen (Siciliano et al., 2017), and the Addenbrooke’s Cognitive Examination (Mioshi et al., 2006)] which inherently rely on motor functions becomes increasingly problematic. The oculomotor control pathways are apparently resistant to pathological degeneration in MND (Smith and Crawford, 2021). As such, abnormalities in oculomotor functions largely driven by cognitive operations (including working memory, inhibitory control) involved in the control of antisaccades, memory guided saccades and the focused attention required for smooth pursuit eye movements, may potentially be reflective of specific frontotemporal cognitive deficits, in particular executive function, attentional processes and inhibitory control, in MND rather than alternations in oculomotor neurons. Thus, SEM and smooth pursuit eye movements may prove to be ideal mechanistic markers of specific cognitive functions in MND. To explore the scope for saccadic and smooth pursuit eye movements to serve as potential markers of specific cognitive function in MND, this review sought to summarize the latest developments in the literature concerning the high level cognitive control of saccadic and standard smooth pursuit eye movement task performance in people with MND.

Although the prosaccade task was the most frequently employed eye tracking task (*n* = 19), the results yielded from this task were perhaps the most inconsistent. Specifically, 52.6% (*n* = 10) of studies observed that prosaccade eye movement metrics in people with MND were comparable to controls; the remaining 47.4% (*n* = 9), however, observed that prosaccades in people with MND differed from controls in terms of duration (Witiuk et al., 2014), latency (Marti-Fàbregas and Roig, 1993; Shaunak et al., 1995; Evdokimidis et al., 2002; Gorges et al., 2015; Rekik et al., 2022; Zaino et al., 2023) velocity (Gorges et al., 2015; Raveh et al., 2023), and the overall occurrence of abnormalities (Poletti et al., 2021). Further inconsistencies were observed when investigating the influence of clinical manifestation and disease severity on prosaccade metrics. Therefore, it appears that the standard prosaccade task may perhaps not be sufficiently specific and sensitive to differentiate people with MND from controls.

The antisaccade task, on the other hand, appears to yield more consistent observations. Specifically, 79% of studies observed characteristic deficits in antisaccade metrics in people with MND compared to controls. People with MND demonstrated elevated antisaccade error rate (Shaunak et al., 1995; Sharma et al., 2012; Witiuk et al., 2014; Gorges et al., 2015; Proudfoot et al., 2016; Becker et al., 2019; Yunusova et al., 2019; Rekik et al., 2022; Raveh et al., 2023; Zaino et al., 2023) and an increase in antisaccade latency (Shaunak et al., 1995; Donaghy et al., 2010; Sharma et al., 2012; Proudfoot et al., 2016). Successful performance on the antisaccade task is assumed to be contingent upon cognitive operations such as executive functioning, that includes working memory, attentional processes, and inhibitory control (Hutton and Ettinger, 2006; Crawford et al., 2011). Therefore, the degree of consistency between observations suggests that the antisaccade task may be sufficiently sensitive to serve as an indicator of these specific cognition functions in MND. Strengthening this assumption, Behler et al. (2021) (which was not included in this review due to the lack of inclusion of a symptomatic MND group) observed that asymptomatic C9orf72 carriers made more antisaccade errors than healthy controls (non-gene carriers), thus, suggesting that antisaccade task metrics may serve as a useful indicator of specific cognitive functions in the asymptomatic/earliest stages of MND.

While the consistency of observations employing the antisaccade task may lead one to assume that the antisaccade task may be an ideal candidate to serve as an indicator of specific cognitive function in MND, it is important to note that no studies have directly compared performance on the antisaccade task to traditional cognitive assessments. Thus, further studies are required before we can confidently extrapolate such a link, and clinical recommendations are made.

Relatively few studies have directly considered the influence of MND subtype on antisaccade metrics, thus permitting firm conclusions regarding the potential application of the antisaccade task in subtyping in MND. However, studies that did consider MND subtype, observed that people with PLS produce a higher error rate and prolonged antisaccade latencies compared to people with ALS (Sharma et al., 2012; Proudfoot et al., 2016). Therefore, the antisaccade task does appear to be sensitive to MND subtype. However, the findings regarding the influence of disease severity and duration are less reliable. Specifically, Proudfoot et al. (2016) observed that disease severity, but not disease duration or rate of progression, were related to antisaccade error rate. In comparison, Zaino et al. (2023) observed that disease duration did significantly influence antisaccade metrics. Therefore, whilst it appears that the antisaccade task may be a useful marker of specific cognitive functions (executive functioning, attentional processes and inhibitory control) in MND, further research is required to reliably ascertain the nuances of this marker.

Only four studies (Evdokimidis et al., 2002; Gorges et al., 2015; Becker et al., 2019; Zaino et al., 2023) analyzed performance of people with MND on the memory guided saccade task. Thus, it appears inappropriate to draw conclusions on the potential utility of the memory guided saccade as a marker of specific cognitive functions in MND. That being said, oculomotor abnormalities, including increased error rate (Gorges et al., 2015; Becker et al., 2019; Zaino et al., 2023) and prolonged latencies (Evdokimidis et al., 2002) were consistently observed during the memory guided saccade task. Thus, should additional further studies corroborate the assumptions of these four studies (Gorges et al., 2015; Kohl et al., 2018; Becker et al., 2019; Zaino et al., 2023), it may be that memory guided saccade metrics may prove a useful marker of specific cognitive functions in MND.

The smooth pursuit task also appears to yield promising consistent results. Specifically, all bar one study observed substantial group level deficits in smooth pursuits in people with MND compared to controls (Abel et al., 1992, 1995; Marti-Fàbregas and Roig, 1993; Donaghy et al., 2010; Moss et al., 2012; Poletti et al., 2021; Guo et al., 2022; Rekik et al., 2022; Raveh et al., 2023). Importantly, the one study, Gorges et al. (2015) that did not observe group level deficits in smooth pursuit eye movements, did however, observe atypicalities at the individual level, indicating that people with MND can potentially be differentiated from controls based on their smooth pursuit task metrics. Smooth pursuit eye movements have traditionally been viewed as a reflexive behaviors predominantly driven by visual motion signals and mediated by pathways that connect visual areas in the cerebral cortex to motor regions in the cerebellum (Behler et al., 2021). However, recent evidence suggests that this view ought to be reconsidered with several authors positing that the smooth pursuit task depends upon cognitive operations including selection, learning, prediction and attention to environmental motion (Barnes, 2008; Fukushima et al., 2013). In light of this stance, given the consistency in observations the smooth pursuit task may prove an appropriate marker of these specific cognitive functions in MND. Further buttressing this assumption, Rekik et al. (2022) observed that the occurrence of abnormal smooth pursuits was correlated with cognitive dysfunction, as classified by the Mini Mental State Examination (Folstein et al., 1983) and the Frontal Assessment Battery (Dubois et al., 2000). It is, however, important to note that smooth pursuit dysfunction also appears to correlate with bladder dysfunction (Rekik et al., 2022), bulbar involvement (Guo et al., 2022) rate of disease progression (Marti-Fàbregas and Roig, 1993) and disease severity (Abel et al., 1992). Thus, it may be that smooth pursuit dysfunction relates to disease progression rather than cognitive function *per se*. Therefore, further studies directly correlating smooth pursuit metrics to both neuropsychiatric cognitive assessments and metrics of disease progression are required to elucidate whether smooth pursuit deficits are indicative of disease progression for frontotemporal cognitive function.

Whilst there has been consistent interest in oculomotor function in people with MND, with the earliest paper being published in 1978 and papers being published until the present day, only 22 papers met the inclusion criterion for this review. Therefore, although there appears to be theoretical and clinical interest in oculomotor function in MND, an increase in research efforts in this area is required to reinforce prior assumptions and enable reliable clinically relevant conclusions to made.

In addition to increasing the quantity of investigations in this area, the methodological quality of subsequent investigations could be improved. Assessment of methodological quality revealed that most studies did not recruit a sample representative of the total population of people with MND. Specifically, where MND severity was reported, the ALS Functional Rating Scale-Revised questionnaire score fell in the range of ∼19–39 (out of a possible 48). Whilst conducting studies with patients in the more advanced stages of MND may be difficult, such studies are required to ascertain the utility of saccade and smooth pursuit eye movements as marker of cognitive function across all stages of MND. Moreover, very few studies reported the basic demographic characteristics of the recruited sample, thus it is not possible to assert that saccade and smooth pursuit eye movements are resistant to demographic and socioeconomic factors within the context of MND. Moreover, despite a wealth of evidence demonstrating that variable clinical facets independently influence saccade and smooth pursuit eye movements e.g., (Ainsworth and Garner, 2013; Anderson and MacAskill, 2013; Bittencourt et al., 2013; Bey et al., 2018; Caldani et al., 2020), very few studies acknowledged this body of work and made reasonable adjustments (e.g., controlling for confounds in statistical analyses) to account for the influence of alternative clinical diagnoses. Moreover, despite it being well established that various saccadic and smooth pursuit metrics (e.g., antisaccade error rate and latency) are influenced by age e.g., (Mack et al., 2020; Yang et al., 2024), very few studies acknowledged this by controlling for the age during analysis. Therefore, a certain degree of caution should be applied to the global findings outlined above, and future studies should endeavor to control for the independent influence of alternative clinical diagnoses and age on saccade and smooth pursuit eye movements.

In summary, we echo prior conclusions that the potential for eye tracking as a marker of cognitive function in a wealth of clinical conditions should not be overlooked (Readman et al., 2021). Of the studies reviewed in this paper, noticeable patterns are observed, and this indicates that the antisaccade task and smooth pursuit task may be sufficiently sensitive to serve as an indicator of specific cognitive operations (including working memory and inhibitory control) in MND. This conclusion could perhaps be extended to include the memory guided saccade paradigm, should further studies confirm the assumptions presented here. However, we highlight the need for further studies which focus on ascertaining the concordance between eye tracking measures and more traditional measures of cognition. Furthermore, we advocate for future studies to more carefully consider the influence of potential confounds and strive to recruit more representative samples.

## Data availability statement

The original contributions presented in this study are included in the article/Supplementary material, further inquiries can be directed to the corresponding author.

## Author contributions

MR: Funding acquisition, Conceptualization, Data curation, Investigation, Methodology, Project administration, Writing – original draft. MP: Conceptualization, Data curation, Investigation, Methodology, Writing – review and editing. MG: Methodology, Investigation, Writing – review and editing. AD: Investigation, Writing – review and editing. SC: Conceptualization, Writing – review and editing. TC: Funding acquisition, Conceptualization, Writing – review and editing, Supervision.

## Footnotes

1
http://retractiondatabase.org

## Appendix

**Appendix 1:** Applied search strings.

Database	Search ID	Search string
Academic Search Ultimate	S1	((DE “MOTOR neuron diseases” OR DE “AMYOTROPHIC lateral sclerosis” OR DE “PROGRESSIVE bulbar palsy”)) OR TI ((MND OR “Motor Neur* Disease*” OR ALS OR “Amyotrophic Lateral Sclerosis “OR “Lou Gehrig*” OR “Progressive Muscular Atroph*” OR “Primary lateral Sclerosis” OR PLS)) OR AB ((MND OR “Motor Neur* Disease*” OR ALS OR “Amyotrophic Lateral Sclerosis” OR “Lou Gehrig*” OR “Progressive Muscular Atroph*” OR “Primary lateral Sclerosis” OR PLS))
	S2	(DE “EYE movements” OR DE “EYE movement measurements” OR DE “EYE tracking” OR DE “SACCADIC eye movements” OR DE “EYE tracking”) OR TI (“eye track*” OR “eye-track*” OR Oculomotor OR Ocularmotor OR “memory guided” OR “memory-guided” OR saccad*
	S3	OR pro-saccad* OR prosaccade* OR “pro saccad*” OR anti-saccad* OR antisaccad* OR “anti saccad*” OR ((eye* OR retina* OR ocular* OR optic*) N3 (mov* OR track*))) OR AB (“eye track*” OR “eye-track*” OR Oculomotor OR Ocularmotor OR “memory guided” OR “memoryguided” OR saccad* OR pro-saccad* OR prosaccade* OR “pro saccad*” OR anti-saccad* OR antisaccad* OR “anti saccad*” OR ((eye* OR retina* OR ocular* OR optic*) N3 (mov* OR track*)))
		S1 AND S2
CINHAL	S1	((MH “Motor Neuron Diseases+”) OR (MM “Amyotrophic Lateral Sclerosis”)) OR TI ((MND OR “Motor Neur* Disease*” OR ALS OR “Amyotrophic Lateral Sclerosis” OR “Lou Gehrig*” OR “Progressive Muscular Atroph*” OR “Primary lateral Sclerosis” OR PLS)) OR AB ((MND OR “Motor Neur* Disease*” OR ALS OR “Amyotrophic Lateral Sclerosis” OR “Lou Gehrig*” OR “Progressive Muscular Atroph*” OR “Primary lateral Sclerosis” OR PLS))
	S2	(MM “Eye Movements+”) OR TI (“eye track*” OR “eye-track*” OR Oculomotor OR Ocularmotor OR “memory guided” OR “memoryguided” OR saccad* OR pro-saccad* OR prosaccade* OR “pro saccad*” OR anti-saccad* OR antisaccad* OR “anti saccad*” OR ((eye* OR retina* OR ocular* OR optic*) N3 (mov* OR track*))) OR AB (“eye track*” OR “eye-track*” OR Oculomotor OR Ocularmotor OR “memory guided” OR “memory-guided” OR saccad* OR pro-saccad* OR prosaccade* OR “pro saccad*” OR anti-saccad* OR antisaccad*OR “anti saccad*” OR ((eye* OR retina* OR ocular* OR optic*) N3 (mov* OR track*)))
	S3	S1 AND S2
Medline	S1	((MM “Motor Neuron Disease+”) OR (MM “Amyotrophic Lateral Sclerosis”)) OR TI ((MND OR “Motor Neur* Disease*” OR ALS OR “Amyotrophic Lateral Sclerosis” OR “Lou Gehrig*” OR “Progressive Muscular Atroph*” OR “Primary lateral Sclerosis” OR PLS)) OR AB ((MND OR “Motor Neur* Disease*” OR ALS OR “Amyotrophic Lateral Sclerosis” OR “Lou Gehrig*” OR “Progressive Muscular Atroph*” OR “Primary lateral Sclerosis” OR PLS))
	S2	(MM “Eye Movements+”) OR TI (“eye track*” OR “eye-track*” OR Oculomotor OR Ocularmotor OR “memory guided” OR “memoryguided” OR saccad* OR pro-saccad* OR prosaccade* OR “pro saccad*” OR anti-saccad* OR antisaccad* OR “anti saccad*” OR ((eye* OR retina* OR ocular* OR optic*) N3 (mov* OR track*))) OR AB (“eye track*” OR “eye track*” OR Oculomotor OR Ocularmotor OR “memory guided” OR “memory-guided” OR saccad* OR pro-saccad* OR prosaccade* OR “pro saccad*” OR anti-saccad* OR antisaccad* OR “antisaccad*” OR ((eye* OR retina* OR ocular* OR optic*) N3 (mov* OR track*)))
	S3	S1 AND S2
Web of Science	S1	(TI =((MND OR “Motor Neur* Disease*” OR ALS OR “Amyotrophic Lateral Sclerosis” OR “Lou Gehrig*” OR “Progressive Muscular Atroph*” OR “Primary lateral Sclerosis” OR PLS))) OR AB =((MND OR “Motor Neur* Disease*” OR ALS OR “Amyotrophic Lateral Sclerosis” OR “Lou Gehrig*” OR “Progressive Muscular Atroph*” OR “Primary lateral Sclerosis” OR PLS))
	S2	(TI =((“eye track*” OR “eye-track*” OR Oculomotor OR Ocularmotor OR “memory guided” OR “memory-guided” OR saccad* OR pro-saccad* OR prosaccade* OR “pro saccad*” OR anti-saccad* OR antisaccad* OR “anti saccad*” OR ((eye* OR retina* OR ocular* OR optic*) N3 (mov* OR track*))) OR AB (“eye track*” OR “eye-track*” OR Oculomotor OR Ocularmotor OR “memory guided” OR “memory-guided” OR saccad* OR pro-saccad* OR prosaccade* OR “pro saccad*” OR anti-saccad* OR antisaccad* OR “anti saccad*” OR ((eye* OR retina* OR ocular* OR optic*) N3 (mov* OR track*))))
	S3	S1 AND S2
Scopus	S1	TITLE (mnd OR “motor neur* disease*” OR als OR “amyotrophic lateral sclerosis” OR “lou gehrig*” OR “progressive muscular atroph*” OR “primary lateral sclerosis” OR pls) OR ABS (mnd OR “motor neur* disease*” OR als OR “amyotrophic lateral sclerosis” OR “lou gehrig*” OR “progressive muscular atroph*” OR “primary lateral sclerosis” OR pls)
	S2	TITLE (”eye track*” OR “eye-track*” OR oculomotor OR ocularmotor OR “memory guided” OR “memory-guided” OR saccad* OR pro-saccad* OR prosaccade* OR “pro saccad*” OR anti-saccad* OR antisaccad* OR “anti saccad*” OR ((eye* OR retina* OR ocular* OR optic*) W/3 (mov* OR track*))) OR ABS (”eye track*” OR “eye-track*” OR oculomotor OR ocularmotor OR “memory guided” OR “memory-guided” OR saccad* OR pro saccad* OR prosaccade* OR “pro saccad*” OR antisaccad* OR antisaccad* OR “anti saccad*” OR ((eye* OR retina* OR ocular* OR optic*) W/3 (mov* OR track*)))
	S3	S1 AND S2
