# Supplementary material for: Oculomotor atypicalities in motor neurone disease: a systematic review
Source: Front Neurosci. 2024 Jun 26;18:1399923. doi: 10.3389/fnins.2024.1399923 (PMC11233471; doi:10.3389/fnins.2024.1399923)
Supplement: Supplementary file 2 [file Table_2.docx]

Supplementary Material 2

# Methodological quality assessment

Table S2. Quality assessment ratings by paper

| **Authors** | Are there clear research questions? | Do the collected data allow to address the research questions? | Are the participants representative of the target population? | Are measurements appropriate regarding both the outcome and intervention (or exposure)? | Are there complete outcome data? | Are the confounders accounted for in the design and analysis? | Is the study administered as intended? |
| --- | --- | --- | --- | --- | --- | --- | --- |
| Sharma et al. [38] | No | Yes | No | Yes | No | No | Yes |
| Proudfoot et al. [39] | Yes | Yes | No | Yes | No | No | Yes |
| Guo et al. [41] | Yes | Yes | No | Yes | Yes | Yes | Yes |
| Yunusova et al.[49] | Yes | Yes | No | No | Yes | No | Yes |
| Moss et al. [50] | No | Yes | Yes | No | Yes | No | Yes |
| Zaino et al. [51] | Yes | Yes | Yes | Yes | Yes | No | Yes |
| Gorges et al. [52] | Yes | Yes | No | Yes | Yes | No | Yes |
| Evdokimidis et al. [53] | Yes | Yes | No | Yes | Yes | No | Yes |
| Burrell et al. [54] | Yes | Yes | No | Yes | Yes | No | Yes |
| Becker et al. [55] | Yes | Yes | No | Yes | Yes | No | Yes |
| Shaunak et al. [56] | Yes | Yes | No | Yes | Yes | No | Yes |
| Rekik et al. [57] | Yes | Yes | No | Yes | No | No | Yes |
| Witiuk et al. [58] | Yes | Yes | No | Yes | No | No | Yes |
| Riek et al.[59] | Yes | Yes | Yes | Yes | No | No | Yes |
| Raveh et al. [60] | Yes | Yes | No | Yes | Yes | No | Yes |
| Poletti et al. [61] | Yes | Yes | No | No | Yes | No | Yes |
| Marti-Fàbregas et al. [62] | No | Yes | No | Yes | Yes | No | Yes |
| Donaghy et al. [63] | No | Yes | No | Yes | Yes | Yes | Yes |
| Abel et al. [64] | Yes | Yes | No | Yes | No | No | Yes |
| Abel et al. [65] | Yes | Yes | No | Yes | No | No | Yes |
| Anagnostou et al.[66] | Yes | Yes | No | Yes | Yes | No | Yes |
| Anagnostou et al. [67] | Yes | Yes | No | Yes | No | No | Yes |
| Esteban et al. [68] | No | Yes | No | Yes | No | No | Yes |
